# Occurrence and characterization of β-lactamase-producing bacteria in biomedical wastewater and *in silico* enhancement of antibiotic efficacy

**DOI:** 10.3389/fmicb.2023.1292597

**Published:** 2024-01-08

**Authors:** Sultana Juhara Mannan, Shopnil Akash, Sumaya Afnan Jahin, Ahnaf Tahmid Saqif, Kohinur Begum, Mahmuda Yasmin, Chowdhury Rafiqul Ahsan, Baye Sitotaw, Turki M. Dawoud, Hiba-Allah Nafidi, Mohammed Bourhia

**Affiliations:** ^1^Department of Microbiology, University of Dhaka, Dhaka, Bangladesh; ^2^Department of Pharmacy, Faculty of Allied Health Sciences, Daffodil International University, Dhaka, Bangladesh; ^3^Department of Microbiology, Jashore University of Science and Technology, Jessore, Bangladesh; ^4^Stamford University Bangladesh, Dhaka, Bangladesh; ^5^Department of Pharmacy, State University, Dhaka, Bangladesh; ^6^Department of Biology, Bahir Dar University, Bahir Dar, Ethiopia; ^7^Department of Botany and Microbiology, College of Sciences, King Saud University, Riyadh, Saudi Arabia; ^8^Department of Food Science, Faculty of Agricultural and Food Sciences, Laval University, Quebec City, QC, Canada; ^9^Department of Chemistry and Biochemistry, Faculty of Medicine and Pharmacy, Ibn Zohr University, Laayoune, Morocco

**Keywords:** hospital effluents, antibiotic-resistant, β-lactamase diversity, molecular docking, binding affinity, ESBL-producing genes

## Abstract

Wastewater discharged from hospitals is a recognized contributor to the dissemination of antibiotic-resistant bacteria and their associated genetic traits into the environment. This study focused on the analysis of β-lactamase-producing pathogenic bacteria within untreated biomedical wastewater originating from various hospitals in Dhaka City, Bangladesh, as well as *in silico* evaluation and structural activity relationship mentioned antibiotics were evaluated. *In silico* drug design techniques were applied to identify the relationship with how the functional group impacts the binding energy. Out of the 184 isolates obtained from well-established hospital sewage discharge points in Dhaka, 89 were identified as β-lactamase positive. These bacteria were subjected to antimicrobial susceptibility testing using the VITEK-2 assay, and their profiles of extended-spectrum beta-lactamase (ESBL) production were determined through molecular methodologies. Among the β-lactamase-positive isolates, considerable resistance was observed, particularly against ampicillin, Ceftriaxone, Cefuroxime, and Meropenem. The predominant resistant species included *Escherichia coli, Acinetobacter baumannii, Pseudomonas aeruginosa*, and *Enterobacter cloacae*. The study identified the prevalence of ESBL-producing genes, with *blaNDM-1* being the most prevalent, followed by *bla_OXA_-1, blaSHV*, *bla_CTX_-_M_*, and *bla_KPC_*. None of the isolates carried the *bla_TEM_* gene. In addition to characterizing these bacteria, the research explored ways to enhance the binding energy of four existing antibiotics as new inhibitors through computational studies. The findings revealed significant improvements in binding energy. Specifically, Meropenem initially exhibited a binding energy of −7.5 kcal/mol, notably increasing to −8.3 kcal/mol after modification. With an initial binding energy was only −7.9 kcal/mol, Ampicillin experienced an enhancement, reaching −8.0 kcal/mol post-modification. Similarly, Ceftriaxone, with an initial binding energy of −8.2 kcal/mol, increased to −8.5 kcal/mol following structural adjustments. Finally, Cefuroxime, initially registering a binding energy of −7.1 kcal/mol, substantially increased to −8.9 kcal/mol after modification. This finding establishes a foundation for future investigations in the development of modified antibiotics to address the issue of antibiotic resistance. It presents prospective remedies for the persistent problem of antibiotic-resistant bacteria in healthcare and the environment.

## 1 Introduction

In densely populated urban areas like Dhaka City, the management of biomedical effluents poses a serious challenge due to the vast quantities of waste generated by healthcare facilities. Hospital wastewater generally contains a high concentration of biochemical oxygen demand (BOD), chemical oxygen demand (COD), total organic carbon (TOC), ammonia nitrogen, organic nitrogen, nitrites, nitrates, total phosphorus, as well as total solids (Emmanuel et al., 2005; Akin, 2016; Young and Lipták, 2018). In addition, it also acts as a reservoir for a significant concentration of pathogens (bacteria, viruses, protozoa, and fungi), antibiotic-resistance genes (ARGs), and antibiotic-resistant bacteria (Weiner et al., 2016). It has been observed that hospital effluents discharged into the sewer system, typically without pre-treatment, can act as a potential source of antimicrobial residues and antibiotic-resistant bacteria for the wastewater treatment plant (WWTP) (Deguenon et al., 2022). In healthcare settings, the emergence and spread of pathogenic bacteria that produce β-lactamases have become a severe issue because they can cause resistance to commonly used β-lactam antibiotics like penicillin. Multidrug-resistant ESKAPE bacteria and *Escherichia coli* (*E. coli*) cause cephalosporin-resistant infections. These infections are the majority of life-threatening bacterial infections among critically ill and immunocompromised patients worldwide. The ESKAPE bacteria include *Enterococcus* spp., *Staphylococcus aureus, Klebsiella pneumoniae*, *Acinetobacter baumannii*, *Pseudomonas aeruginosa*, and *Enterobacter* spp. Therefore, the current study was primarily done to identify the occurrence and characterize the pathogenic bacteria producing β-lactamase isolated from the biomedical, along with *E. coli* (Weiner et al., 2016). The most remarkable resistance mechanism among Gram-negative bacteria to the selective pressure of antibiotics is caused by β-lactamase enzymes. The genes that confer resistance can be transferred either vertically (to bacterial offspring) or horizontally (among different bacterial species) (Adekunle, 2012; Baquero et al., 2021). ESBL enzymes can be classified into around 300 subtypes, and among them, genes encoding blaCTX, blaTEM, blaOXA, and blaSHV are the most common types (Korzeniewska and Harnisz, 2013). The carbapenemase family of β-lactamases (a distinct subgroup) is one of the most threatening enzymes responsible for the resistance of the aforementioned strains. Several different forms of genes encode carbapenemase found in *Enterobacteriaceae*, including blaKPC and blaNDM-1 (El Salabi et al., 2013).

These enzymes have been found in numerous *Enterobacteria* species that have been isolated in hospitals worldwide. *E. coli* and *Klebsiella pneumoniae* were the first organisms to contain plasmids that encoded the blaNDM-1 gene; nevertheless, it has been demonstrated that these plasmids may be transferred to other species, aiding in the spread of the blaNDM-1 gene to other species as well. The prevalence of β-lactamase enzymes has steadily risen, leading to the reduced efficacy of many commonly prescribed antibiotics (Bush, 2010; Dortet et al., 2014). This phenomenon seriously affects public health, as it limits the treatment options available to medical professionals and increases the risk of infections becoming untreatable. Identifying the presence and understanding of the characteristics of pathogenic bacteria producing β-lactamase in hospital wastewater is of utmost importance to develop effective strategies for combating antibiotic resistance and safeguarding public health (Muteeb et al., 2022a; Irfan et al., 2023).

Moreover, the transmission of multidrug-resistant bacteria has become a severe threat in Bangladesh, as antibiotics can be easily purchased without a prescription from a registered physician (Islam, 2021). As hospital effluents can harbor different pathogens, they can act as reservoirs for superbugs. This study aimed to identify and characterize pathogenic bacteria producing β-lactamase in biomedical wastewater samples from Dhaka City hospitals. Additionally, we employed computational methods to enhance the antibacterial effectiveness of four commonly encountered antibiotics: Meropenem (100% resistance), Ampicillin (80–93% resistance), Ceftriaxone (73–100% resistance), and Cefuroxime (80–95% resistance). These modifications were designed to inhibit the growth of *E. coli* bacteria and reduce the likelihood of antibiotic resistance development in this pathogen.

Since these antibiotics are almost unable to fight against bacteria. Our work utilizes *in silico* approaches to evaluate the effects of functional groups on binding affinity, in light of the inadequate efficacy of Meropenem, Ampicillin, Ceftriaxone, and Cefuroxime against bacteria. β-lactamase-producing bacteria in biomedical wastewater often develop resistance to these antibiotics. Therefore, it is necessary to investigate improved formulations of these antibiotics to improve their effectiveness against bacteria. Our goal is to analyze the structural and chemical factors that affect binding interactions. This analysis will help us identify ways to enhance the therapeutic properties of these antibiotics. By accomplishing so, we can tackle the problem of bacterial resistance and improve their effectiveness in healthcare and environmental settings.

## 2 Materials and methods

### 2.1 Sample collection

A total of 300 sewage samples were collected from the wastewater disposal units of both public and private hospitals in Dhaka City between December 2022 and March 2023. The samples were acquired on weekdays in the morning, from 8: 00 AM to 11: 00 AM, and stored in 5 mL sterile Falcon tubes, each labeled according to its location. During transportation to the laboratory, the samples were kept in an icebox and processed within 2 h upon arrival.

### 2.2 Growth in nutrient agar

Samples obtained from hospitals were streaked onto nutrient agar medium (HiMedia) containing 2 and 4 μg/mL of Meropenem (Opsonin Pharmaceuticals, Bangladesh). Subsequently, they were incubated at 37°C for 24–48 h under aerobic conditions. Colonies that grew under meropenem selection were sub-cultured on nutrient agar and reserved for later stages of the study.

### 2.3 Identification of organisms using VITEK 2

For identification through the VITEK 2 platform, single colonies were streaked onto nutrient agar plates and incubated at 37°C overnight. One to three pure and distinct colonies were selected from each agar plate and suspended in normal saline to prepare an inoculum with an absorbency of approximately 0.5 McFarland standard before use in the assay. Bacterial strains were identified using the VITEK 2 GN ID card. As a positive control, *Enterobacter hormaechei* (ATCC-700323) was employed.

### 2.4 Antibiotic susceptibility test

The antibiotic susceptibility profile of the respective isolates was determined for 19 antimicrobial agents following CLSI guidelines and the manufacturer’s recommendations. The VITEK 2 system with VITEK 2 Cards (AST N280) was utilized for this analysis. *Enterbacter hormaechei* strain LBM 93.03.067 (ATCC-700323), susceptible to all drugs, served as the quality control for the system. The panel (bioMérieux) of 19 tested antibiotics included amikacin, amoxicillin/clavulanic acid, ampicillin, cefepime, cefoperazone/sulbactam, ceftazidime, Ceftriaxone, Cefuroxime, Cefuroxime Axetil, ciprofloxacin, co-trimoxazole, colistin, ertapenem, gentamicin, imipenem, Meropenem, nalidixic acid, nitrofurantoin, piperacillin, and tigecycline. The isolates were categorized as resistant, intermediate, or susceptible based on the minimum inhibitory concentration of the antibiotics, following CLSI guidelines. Bacterial strains displaying resistance to three or more antibiotic classes were classified as multidrug-resistant. These all antibiotic were taken from Opsonin pharmaceuticals, Bangladesh.

### 2.5 Characterization of ESBL and carbapenemase genes

All the confirmed β-lactamase-positive isolates were screened for the presence of molecular determinants of ESBL and carbapenem resistance, including the blaCTX, blaTEM, blaOXA1, blaSHV, blaNDM-1, and blaKPC families. The screening was conducted using a thermal cycler following a published protocol, with minor optimizations made for the primer annealing temperature. The optimal annealing temperature for each primer (Macrogen inc) was determined through a temperature gradient test ranging from 55 to 65°C, as detailed in Table 1.

**Table 1:** Optimization of primer annealing temperatures for ESBL and carbapenemase genes screening.

Target genes	Primer	Sequences (5′-3′)	Amplicon Length (base pair)	Annealing temperature(°C)	References
*bla*_NDM-1_	*bla*_NDM-1_-forward	AAT GGC TCA TCA CGA TCA TGC	220	60	Haller et al. (2018)
	*bla*_NDM-1_-reverse	GGC CCG CTC AAG GTA TTT TAC			
*bla*_KPC_	*bla*_KPC_ – forward	ATGTCACTGTATCGCCGTCT	893	60	Bratu et al. (2005)
	*bla*_KPC_ – reverse	TTTTCAGAGCCTTACTGC CC			
*bla*_CTX-M_	*bla*_CTX-M_-forward	ATGTGCAGYACCAGTAARGTKATGGC	300	60	Birkett et al. (2007)
	*bla*_CTX-M_ – reverse	ATCACKCGGRTCGCCXGG RAT			
*bla*_SHV_	*bla*_SHV_ – forward	GCCTGTGTATTATCTCCCTGTTAGC	764	60	Soltan et al. (2013)
	*bla*_SHV_ – reverse	CAGATAAATCACCACAATGCGC			
*bla*_OXA-1_	*bla*_OXA-1_- forward	GGCACCAGATTCAACTTTCAAG	564	57.8	Ogutu et al. (2015)
	*bla*_OXA-1_- reverse	GACCCCAAGTTTCCTGTAAGTG			
*bla*_TEM_	*bla*_TEM_ -forward	CATTTCCGTGTCGCCCTTATTC	800	60	Perez et al. (2007)
	*bla*_TEM_ - reverse	CGTTCATCCATAGTTGCCTGAC			

### 2.6 Ligand preparation and optimization

**Figure 1:**
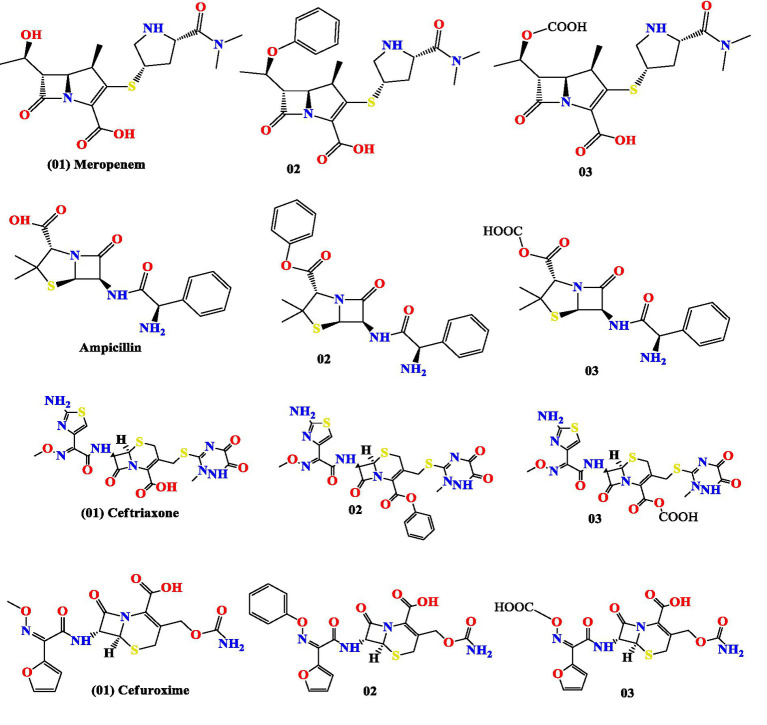
Molecular structure of modified compounds.

The initial structures of Meropenem, Ampicillin, Ceftriaxone, and Cefuroxime were obtained from the PubChem database. Subsequently, we introduced modifications by adding a benzene ring and a -COOH group, as illustrated in Figure 1, using the ChemDraw application. These altered ligand structures were then imported into Gaussian 16 software. For optimization, we employed the hybrid B3LYP exchange-correlation functional in conjunction with the 6-311G(d) basis set, as implemented in Gaussian 16. Semi-core pseudo-potentials were also considered in density-functional theory (DFT) method (Obot et al., 2015; Ribeiro et al., 2015). Finally, the structures were saved in pdb format for subsequent molecular docking analysis.

### 2.7 Protein preparation and molecular docking studies

**Figure 2:**
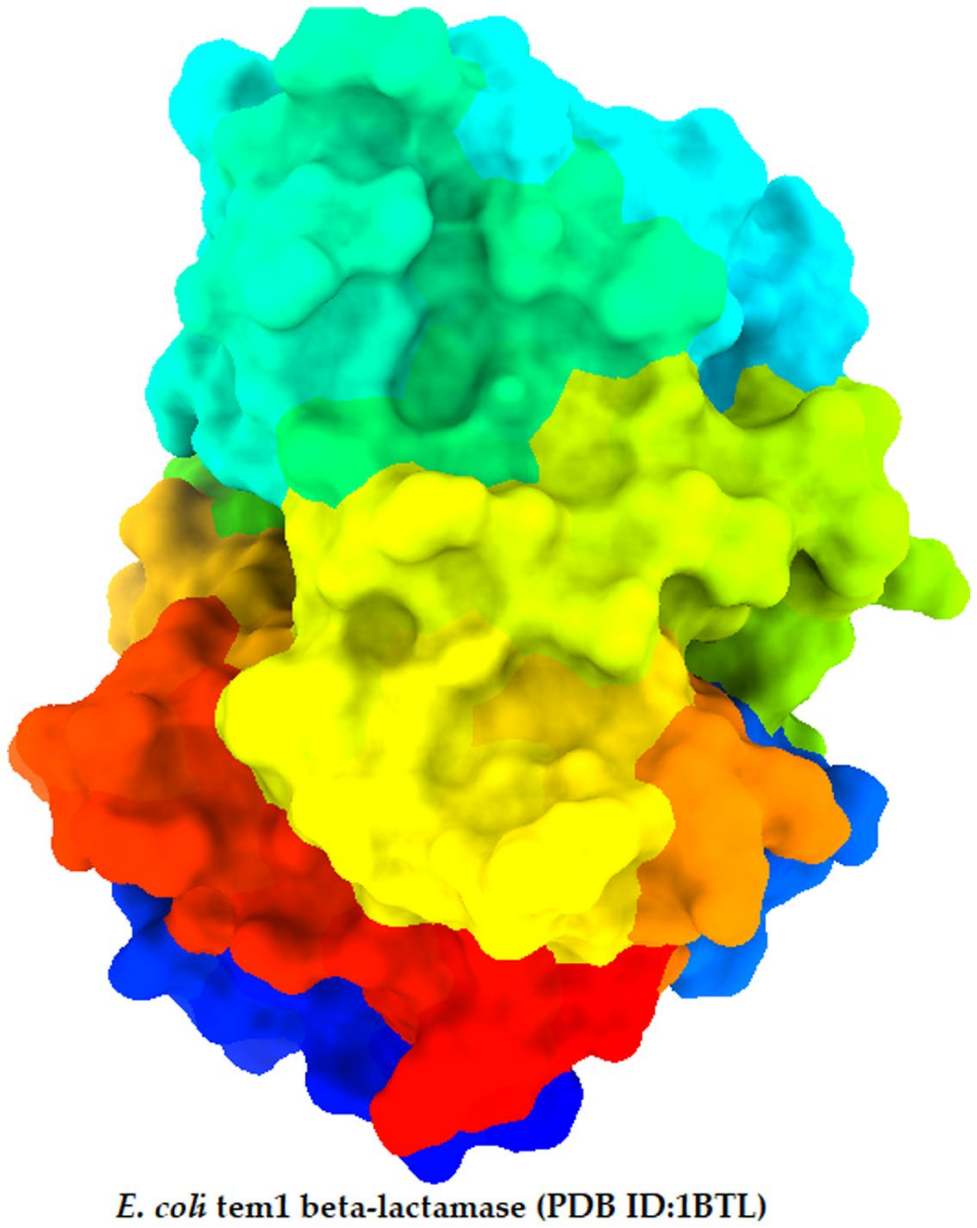
Three-dimensional protein structure of targeted receptor.

Molecular docking analysis is one of the essential method to predict the binding energy of drug protein complexes (Muteeb et al., 2022b). So, in this investigation, we applied molecular docking with our modified ligand and targeted *E. coli* bacterial protein (PDB ID: 1BTL) (Figure 2). Prior to experiment of molecular docking, the protein was acquired in PDB format from the RCSB Protein Data Bank and prepared using Discovery Studio. Subsequently, molecular docking analysis was conducted using PyRx, with grid centre points set at *X* = −11.873884, *Y* = −24.3376, *Z* = 19.0138, and box dimensions specified as *X* = 29.7641, *Y* = 27.76183, *Z* = 28.766. Finally, BIOVIA Discovery Studio Visualizer and ChimeraX were instrumental in visualizing the active amino acid residues (Dallakyan and Olson, 2015; Ahmad et al., 2022).

## 3 Result analysis

### 3.1 Bacterial screening

**Figure 3:**
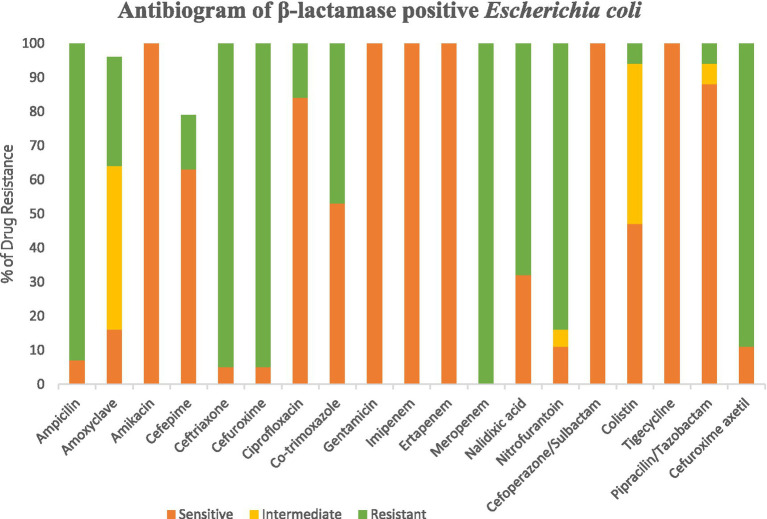
Antimicrobial susceptibility test of the β-lactamase positive strains.

A total of 184 isolated bacterial colonies were obtained under meropenem selection. These isolates were further sub-cultured on nutrient agar medium and identified using VITEK-2.0 system. Of the 184 isolates, 48.4% (*n* = 89) were found to be beta-lactamase positive. The most prominent species identified in hospital effluents throughout the study period were *Escherichia coli* (42.7%, *n* = 38), *Acinetobacter baumanii* (25.9%, *n* = 23), *Pseudomonas aeruginosa* (25.9%, *n* = 23) and *Enterobacter cloacae* (5.6%, *n* = 5) (Figure 3).

### 3.2 Antibiogram of *Acinetobacter baumannii*

**Figure 4:**
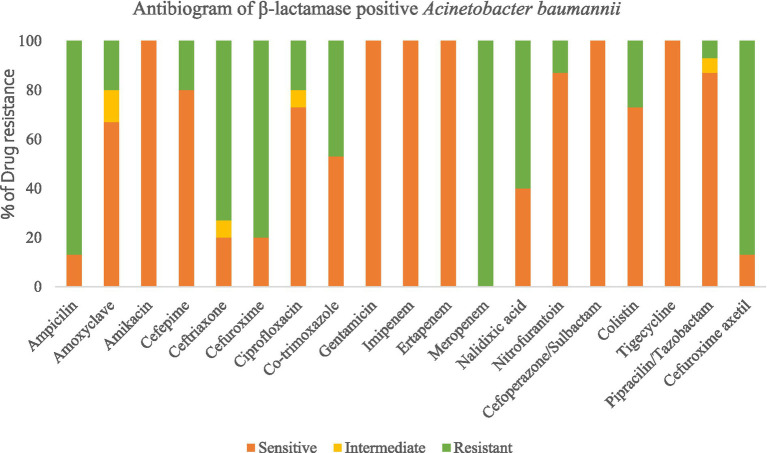
Antibiogram of β-lactamase positive *Acinetobacter baumannii.*

In the case of *A. baumannii*, the highest resistance level was observed against Meropenem, closely followed by ampicillin (with an 87% resistance rate). Notably, all of the isolated strains of this bacterial species exhibited multidrug resistance, as they displayed resistance to antibiotics from three distinct classes (Figure 4).

### 3.3 Antibiogram of *Pseudomonas aeruginosa*

**Figure 5:**
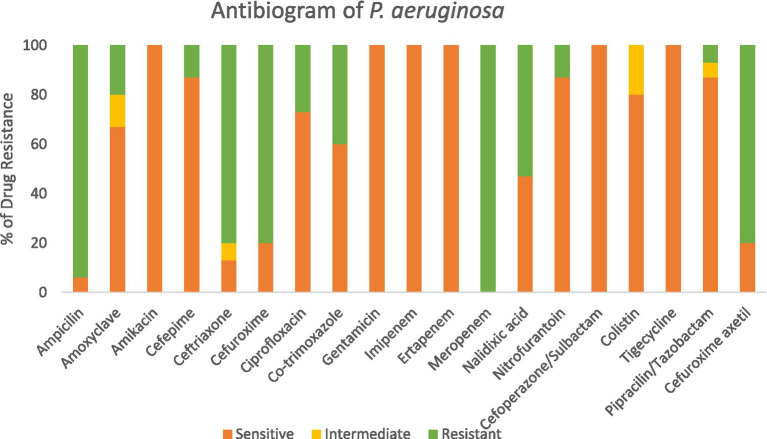
Antibiogram of *P. aeruginosa.*

Meropenem-resistant *Pseudomonas aeruginosa* strains exhibited increased resistance to three distinct antibiotic classes. Among these, the most pronounced resistance was observed against ampicillin, with a resistance rate of 94% (as shown in Figure 5). The resistance patterns against other antibiotics were as follows: Ceftriaxone (80%), Cefuroxime (80%), Nalidixic acid (53%), and Cefuroxime axetil (80%).

### 3.4 Antibiogram of *Enterobacter cloacae*

**Figure 6:**
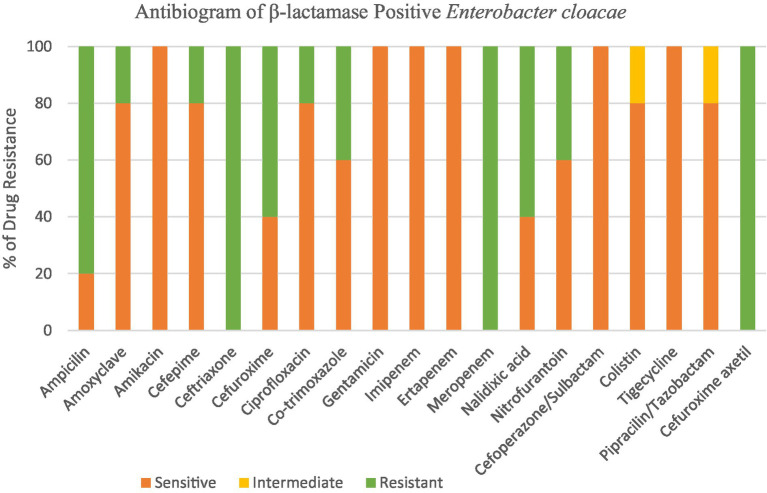
Antibiogram of β-lactamase positive *Enterobacter cloacae.*

Among the five tested *Enterobacter cloacae* isolates, 100% were resistant to Ceftriaxone, Meropenem, and Cefuroxime Axetil, 80% to ampicillin, 60% to Cefuroxime and nalidixic acid, 40% to co-trimoxazole and nitrofurantoin and 20% to ciprofloxacin and amoxiclav. Multidrug resistance (MDR) was detected in all isolates (100%) as shown in Figure 6.

### 3.5 Characterization of ESBL and carbapenemase genes

Among all isolates that tested positive for β-lactamase, an examination was conducted to determine the presence of molecular determinants of ESBL genes. Out of the 89 isolates, the two most predominant carbapenemase genes were *bla*_NDM_-_1_ (100%), followed by *bla*_OXA_ (41.2%). Additionally, other β-lactamase genes, such as as*bla*_SHV_ (5.9%), *bla*_CTX-M_ (5.9%) and *bla*_KPC_ (5.9%), were also detected. Notably, none of the isolates harbored the bla_TEM_ β-lactamase gene.

**Figure 7:**
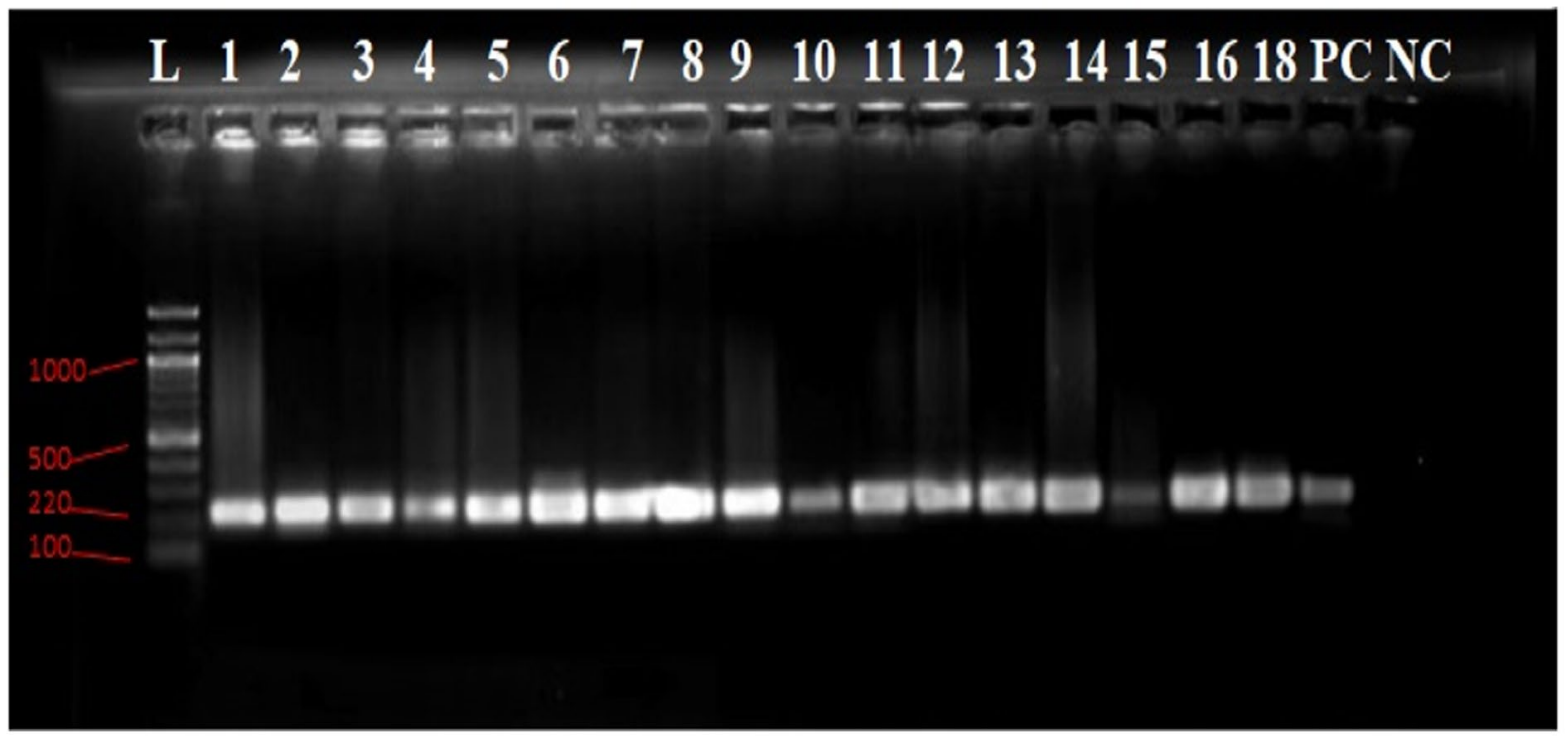
Agarose gel electrophoresis image of the PCR products of *bla*_NDM-1_ gene.

The NDM producers were predominantly found in members of both *Enterobacteriaceae*, such as *E. coli* and *Enterobacter cloacae*, and non-Enterobacteriaceae families, including *Pseudomonas aeruginosa* and *Acinetobacter baumannii*. Furthermore, KPC producers were mainly identified in *Acinetobacter baumannii*, while the OXA gene was predominantly present in *Pseudomonas aeruginosa*. In contrast, the distribution of CTX and SHV was less widespread among the aforementioned species (Figure 7).

**Figure 8:**
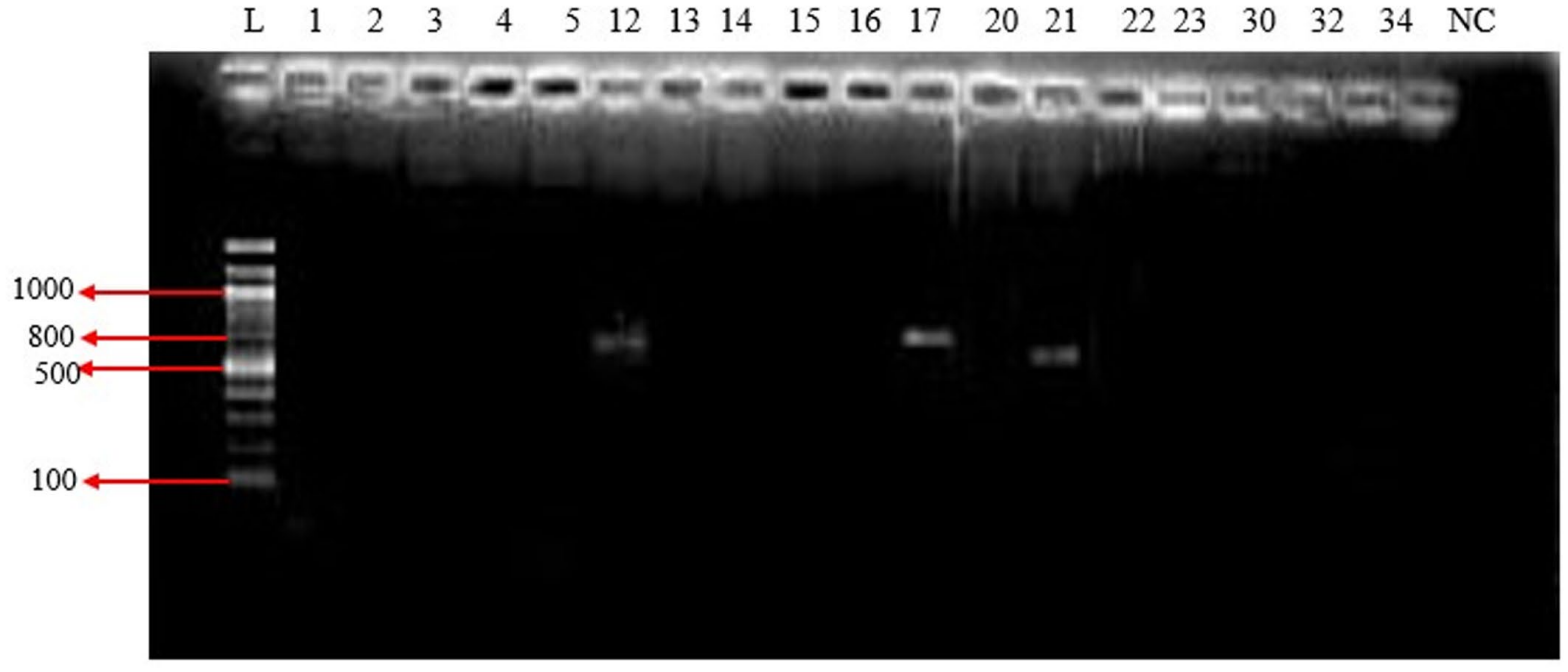
Agarose gel electrophoresis image of the PCR products of *bla*_KPC_ gene.

The gel electrophoresis result shows a molecular weight marker (Gene Ruler 100 bp DNA ladder) in lane L, with the positive control (PC) showing a clear band. Lanes 1 to 16 and 18 exhibit 220 bp PCR product bands, while the negative control (NC-) in lane NC shows no such band (Figure 8).

**Figure 9:**
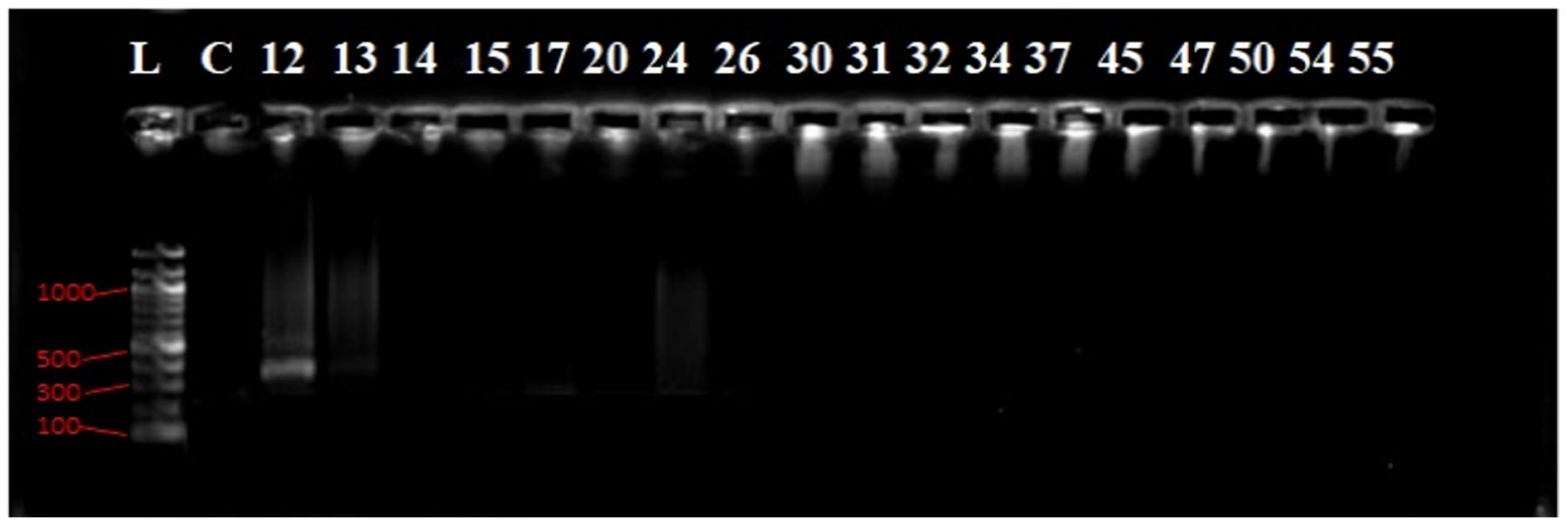
Agarose gel electrophoresis image of the PCR products of *bla*_CTX-M_ GeneL lane, molecular weight marker; Gene Ruler 100 bp DNA ladder, lanes numbered 12 shows (300 bp) bands of PCR products while lane NC show negative control.

In Figure 9, lane L contains a molecular weight marker (Gene Ruler 100 bp DNA ladder). Notably, lanes 12, 17, and 21 display distinct 893 bp PCR products.

**Figure 10:**
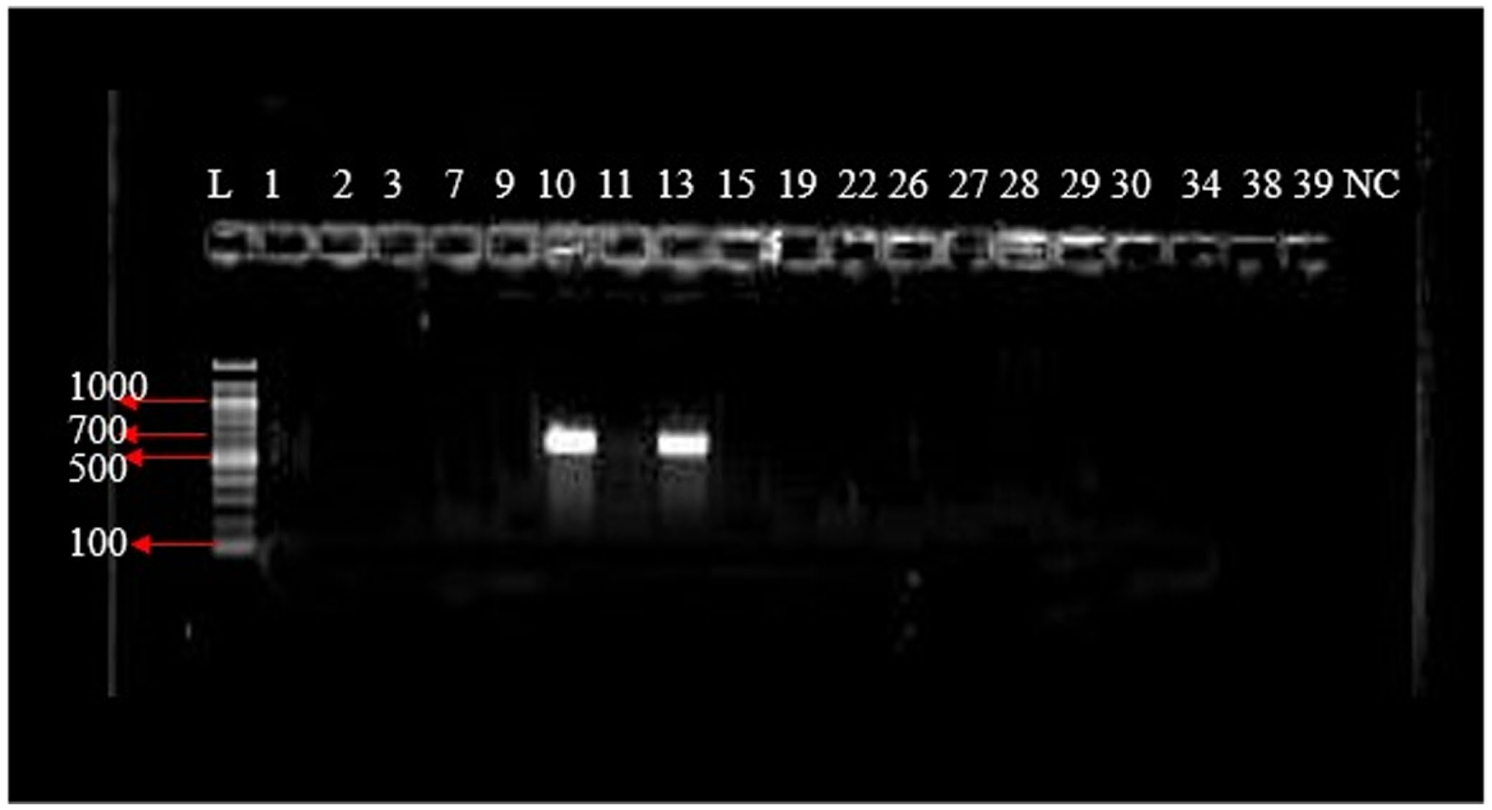
Agarose gel electrophoresis image of the PCR products of *bla*_SHV_ gene.

In Figure 10, Lane 10 and 13 reveal 764 bp PCR product bands, while lane NC- represents the negative control.

**Figure 11:**
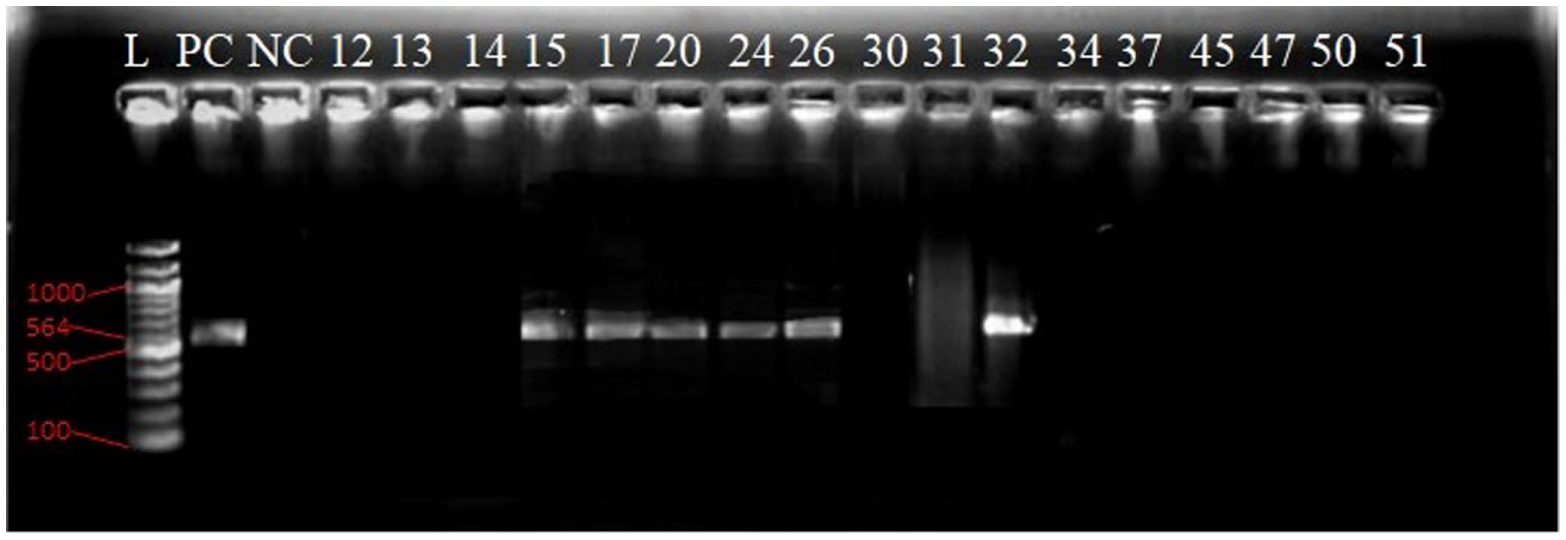
Agarose gel electrophoresis image of the PCR products of *bla*_OXA-1_ gene.

The gel electropherogram shows a molecular weight marker (Gene Ruler 100 bp DNA ladder) in lane L. The positive control (PC) and PCR products in lanes 15, 17, 20, 24, 26, and 32 exhibit 564 bp bands (Figure 11).

### 3.6 Molecular docking against *Escherichia coli*

The molecular docking investigation aimed to determine drug-protein complexes’ binding affinity and active site. In this study, Meropenem (100%), Ampicillin (80–93%), Ceftriaxone (73–100%), and Cefuroxime (80–95%) exhibited the highest levels of resistance against *E. coli*. Consequently, these four established drugs underwent structural modifications and molecular docking experiments.

The results revealed notable changes in binding energy. Specifically, Meropenem exhibited a binding energy of −7.5 kcal/mol, which increased significantly to −8.3 kcal/mol after modification. Ampicillin showed a binding energy of −7.9 kcal/mol, which improved to −8.0 kcal/mol post-modification. Similarly, Ceftriaxone had an initial binding energy of −8.2 kcal/mol, which rose to −8.5 kcal/mol following structural modification. Lastly, Cefuroxime initially had a binding energy of −7.1 kcal/mol, which substantially increased to −8.9 kcal/mol after modification (Table 2).

**Table 2:** Binding affinities of docked ligand calculated against targeted proteins.

*E. coli* tem1 beta-lactamase (PDB ID:1BTL)							
Binding energy Kcal/mol		Binding energy Kcal/mol		Binding energy Kcal/mol		Binding energy kcal/mol	
Meropenem	−7.5	Ampicillin	−7.9	Ceftriaxone	−8.2	Cefuroxime	−7.1
Derivatives 02	−8.3	Derivatives 02	−8.2	Derivatives 02	−7.7	Derivatives 02	−8.9
Derivatives 03	−8.0	Derivatives 03	−8.6	Derivatives 03	−8.5	Derivatives 03	−8.1

These findings underscore the potential for structural enhancements to boost the binding affinity of these antibiotics, thereby offering promising strategies to combat antibiotic resistance effectively.

### 3.7 Active residue analysis and binding pocket

**Figure 12:**
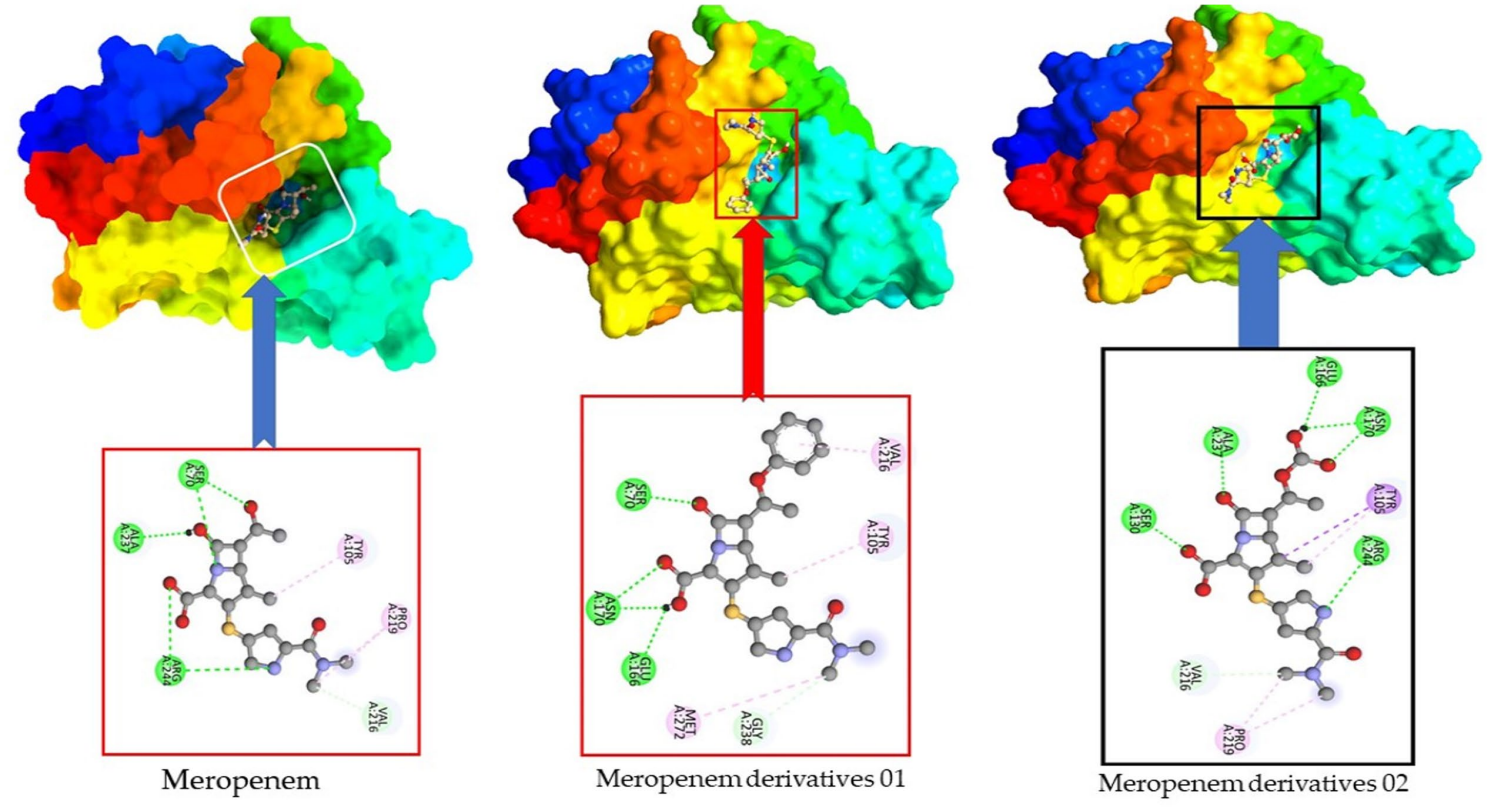
Active site and amino acid residue of Meropenem and its derivatives.

We conducted an analysis of the active residues within the binding pocket to unveil the potential modes and specific amino acids involved in the formation of drug- protein complex complexes. In Figure 12, it becomes evident that the primary Meropenem featured only five active amino acid residues. However, upon the addition of functional groups, the number of active amino acid residues increased significantly. This augmentation in active amino acid residues led to the establishment of robust bonds between *E. coli* and Meropenem derivatives.

This observation highlights the dynamic nature of drug-protein interactions, particularly the impact of functional group additions on the binding characteristics. The increased active amino acid residues likely contribute to enhanced binding affinity, which could have implications for the efficacy and specificity of Meropenem derivatives in combating *E. coli* infections. These findings underscore the importance of the addition of functional group for improved the binding affinity.

**Figure 13:**
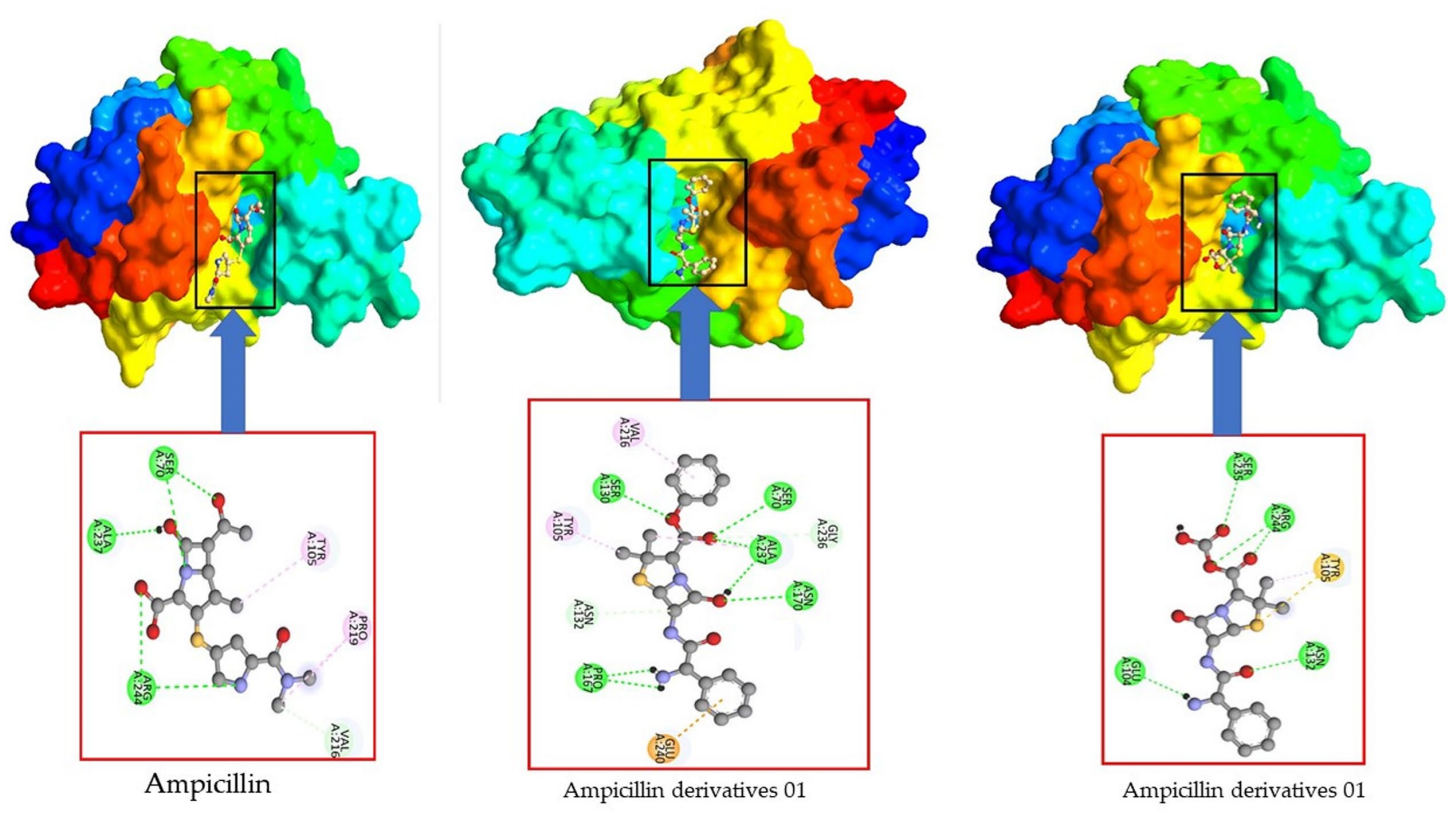
Active site and amino acid residue of ampicillin and its derivatives.

Our investigation revealed that the original ampicillin encompassed just six active amino acid residues inside its binding region. However, when introducing a benzene ring in derivatives 02, the count of active amino acid residues detected a substantial rise, reaching a total of 11. Consequently, the incorporation of the benzene ring increased the antibiotic’s affinity for its target. Interestingly, the addition of other functional groups in derivatives 03 resulted in a decrease in the number of active residues, reducing it to five active sites (as shown in Figure 13).

This observation underscores the dynamic nature of ampicillin and its derivatives in interacting with their target proteins. The changes in active amino acid residues upon structural modifications can have important implications for their binding affinity and, consequently, their effectiveness as antibiotics.

**Figure 14:**
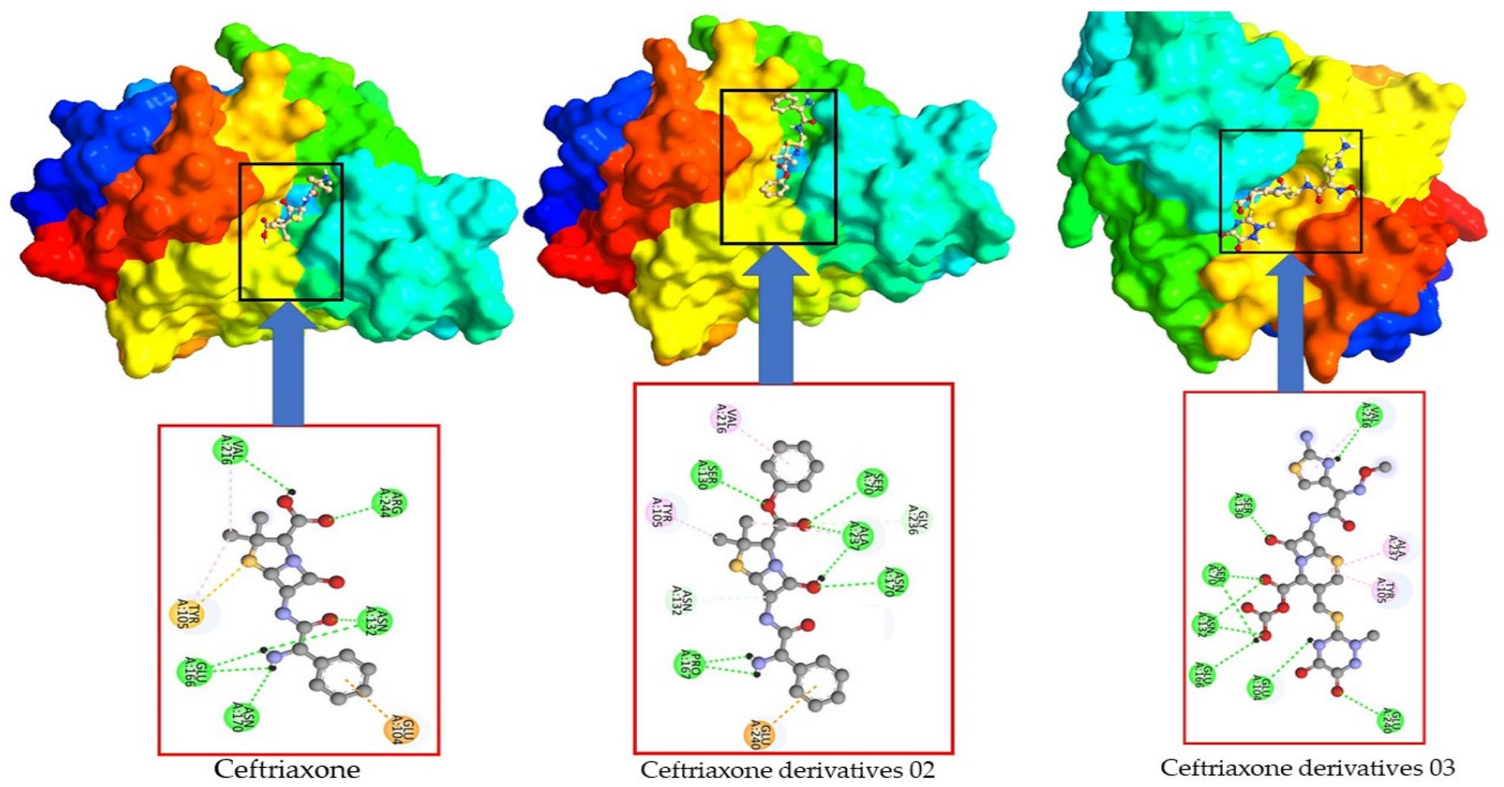
Active site and amino acid residue of Ceftriaxone and its derivatives.

In the case of Ceftriaxone and its derivatives, the primary Ceftriaxone molecule exhibits only seven active amino acid residues. However, upon adding a benzene ring and a COOH functional group, the active site expands to encompass 10 residues in the case of derivatives 02 and 09, as illustrated in Figure 14.

**Figure 15:**
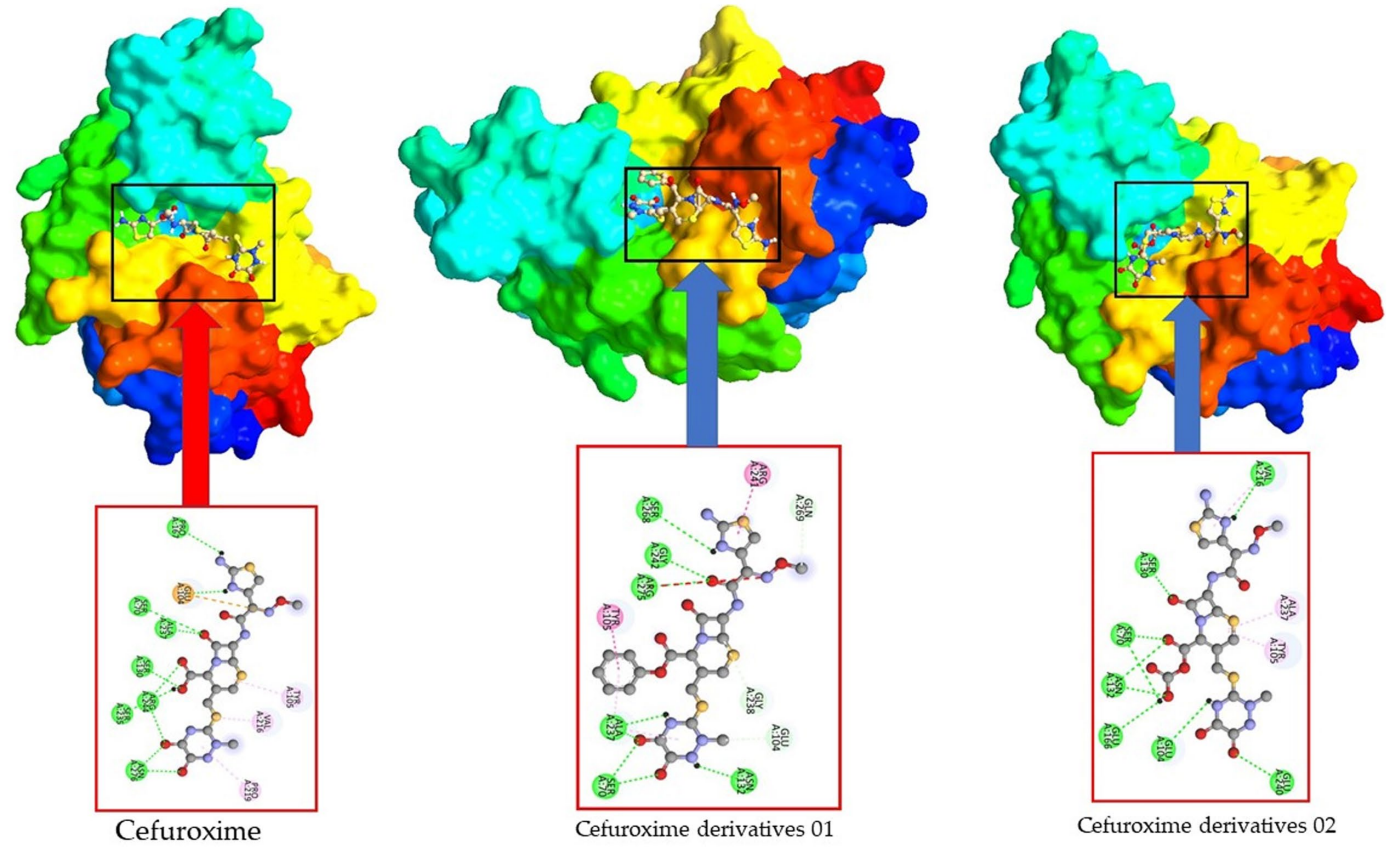
Active site and amino acid residue of Cefuroxime and its derivatives.

In the context of Cefuroxime and its derivatives, there’s a notable observation regarding active amino acid residues. In the case of the primary Cefuroxime molecule, it exhibits a total of 11 active amino acid residues. Interestingly, when we examine derivatives 02, they also demonstrate the same number of active amino acid residues as the primary ligand, which is 11. However, for derivatives 03, there is a slight reduction in the active site, with 10 amino acid residues being involved (as shown in Figure 15).

## 4 Discussion

According to the literature, hospital effluents have been identified as a possible repository for high concentrations of antimicrobials and microorganisms carrying antimicrobial resistance determinants (Chagas et al., 2011). As the -harboring ESBL can hydrolyze a wide variety of β-lactam antibiotics, including penicillin and cephalosporins, they pose significant hurdles in clinical settings, making the increased incidence of these bacteria a worrying global health concern. According to the findings of this study, there is a high burden of ESBL-producing bacteria in hospital effluents, underscoring the crucial role that healthcare facilities play in the spread of antibiotic resistance. Hospital, may act as hub for the emergence and dissemination of these resistant pathogens, given the high prevalence of ESBL-positive isolates in this environment. Moreover, hospital effluents may contain ESBL pathogens for several reasons, including poor disposal of patient waste that contains antibiotic residues, ineffective wastewater treatment methods, and the transfer of resistant bacteria from patients to the hospital environment. *During our study, E. coli was found to be the most common β-lactamase lactamase-producing species in hospital wastewater*.

All four bacterial species displayed the highest levels of resistance to MMeropenem (100%), ampicillin (80–93%), Ceftriaxone (73–100%), and Cefuroxime (80–95%), according to the AMR results analysis. Resistance patterns varied amongst isolates; however, *E. coli* isolates showed the highest levels of resistance, and this finding correlated with previous reports (Rabbani et al., 2017). Furthermore, it was discovered that *E. coli* has developed resistance to the antibiotic colistin, which is used as a last resort antibiotic. In previous research, urban sludge samples from Dhaka, Bangladesh, were shown to have colistin-resistant *E. coli* (Islam et al., 2017). On the contrary, considering these four bacterial species, tigecycline, gentamicin, imipenem, ertapenem, and cefotaxime/sulbactam remained the most effective antibiotics as the isolated strains were susceptible to these antibiotics. Increased levels of *Enterobacteriaceae*-producing carbapenemase have been seen in recent years in both clinical and community settings (Nordmann et al., 2009). While *bla*_NDM-1_ was previously exclusively found in *E. coli* and *Klebsiella pneumoniae*, current investigations have shown it in various organisms, which is concerning (Walsh et al., 2011). The studies carried out in China and Singapore have similar findings regarding intestinal bacteria having high levels of *bla*_NDM-1_in clinical samples (Wang et al., 2013).

Both Enterobacteriaceae (*Escherichia coli*, *Enterobacter cloacae*) and Non- Enterobacteriaceae (*Pseudomonas aeruginosa*, *Acinetobacter baumannii*) were shown to have bla_NDM-1_ in our investigation. Till now, the Enterobacteriaceae group and *Pseudomonas aeruginosa* have been the primary source of bla_KPC_ in clinical settings. However, bla_KPC_ gene was absent in these mentioned bacterial species of our study. In contrast, bla_KPC_ was present only in *Acinetobacter baumannii* species. The occurrence of bla_KPC_ producing isolates from environment are increasing day by day and they have been recovered from various sources.

Furthermore, this investigation conducted *in silico* experiments on four highly resistant antibiotics: Meropenem, Ampicillin, Ceftriaxone, and Cefuroxime, with the aim of enhancing their efficacy and ultimately reducing the likelihood of antibacterial resistance. The findings revealed that initially, the binding affinity was significantly lower in each case. However, upon the addition of functional groups, the binding energy gradually increased. In most instances, the benzene ring played a pivotal role in augmenting the binding energy, although the COOH group was also introduced into these four antibiotics.

These results suggest promising strategies for structural modifications to bolster the effectiveness of these antibiotics, thereby offering potential solutions in the ongoing battle against antibiotic resistance. By improving the binding affinity and energy, these modified antibiotics may exhibit enhanced antimicrobial properties, reducing the risk of bacterial resistance development. This *in silico* approach opens up new avenues for research into combating antibiotic-resistant pathogens and underscores the importance of innovative methods in the field of antibiotic development.

## 5 Conclusion

In summary, this study was conducted to determine the occurrence and characterization of β-Lactamase-producing Bacteria in Biomedical Wastewater and modified Meropenem, Ampicillin, Ceftriaxone, and Cefuroxime to enhance efficacy of mentioned antibiotics using *in silico* studies. It is noted that the alarming presence and attributes of pathogenic β-lactamase-producing bacteria in biomedical wastewater samples collected from hospitals in Dhaka City. The detection of these bacteria, along with their antibiotic resistance profiles, underscores a significant public health concern. These pathogens have the potential to disseminate antibiotic resistance genes within the environment, thus compromising the efficacy of β-lactam antibiotics.

These findings emphasize the critical need for robust wastewater management strategies and stringent infection control measures within healthcare facilities to curb the spread of these resistant pathogens.

Furthermore, we also performed computational investigation and improved the efficacy of these mentioned antibiotics so that possible new antibacterial agent could be developed. Besides, the policymakers and healthcare administrators should prioritize the implementation of rigorous infection control policies and the pre-treatment of effluents before releasing them into the environment. This concerted approach is essential to combat the emergence and transmission of multidrug-resistant and ESBL-producing bacteria. In essence, this research underscores the significance of proactive measures in safeguarding public health and combating antibiotic resistance, particularly in densely populated urban areas like Dhaka City, where the management of biomedical effluents presents a significant challenge.

## Data availability statement

The original contributions presented in the study are included in the article/supplementary material, further inquiries can be directed to the corresponding authors.

## Author contributions

SJM: Conceptualization, Investigation, Writing – original draft, Data curation, Methodology. SA: Conceptualization, Investigation, Writing – original draft, Methodology, Validation. SAJ: Methodology, Writing – original draft, Data curation, Validation. ATS: Writing – original draft, Data curation, Writing – review & editing. KB: Data curation, Project administration, Resources, Validation, Writing – review & editing. MY: Conceptualization, Formal analysis, Supervision, Resources, Writing – review & editing. CRA: Formal analysis, Project administration, Writing – original draft. BS: Investigation, Conceptualization, Writing – original draft, Writing – review & editing, Funding acquisition. TMD: Investigation, Methodology, Writing – review & editing. H-AN: Supervision, Project administration, Funding acquisition. MB: Investigation, Data curation, and Project administration.
